# Supplementary material for: Active School Commuting in School Children: A Narrative Review of Current Evidence and Future Research Implications
Source: Int J Environ Res Public Health. 2023 Oct 16;20(20):6929. doi: 10.3390/ijerph20206929 (PMC10606062; doi:10.3390/ijerph20206929)
Supplement: Supplementary file 1 [file ijerph-20-06929-s001.zip › ijerph-2631963-supplementary.pdf]

Table S1 Information of papers included in the review.

Author and year of publication	Reference	Article type	Country/Region	Key / relevant findings
1. What are the trends of the global prevalence of ASC?				
Grize et al. 2010	[14]	Longitudinal study	Switzerland	Proportion of children walked or cycled to school dropped from 78.4% to 71.4% between 1994 and 2005.
Black, Collins & Snell 2016	[15]	Longitudinal study	United Kingdom	Proportion of children walked or cycled to school dropped from 73% to 63% between 1975 and 1994.
McDonald 2007	[16]	Longitudinal study	United States	Proportion of children walked or cycled to school dropped from 40.7% to 12.9% between 1969 and 2001
Pavelka et al. 2017	[18]	Longitudinal study	Czech Republic	Proportion of children walked or cycled to school dropped from 74.3% to 53.4% between 2006 and 2014.
Gálvez-Fernández et al. 2021	[22]	Longitudinal study	Spain	ASC rates among children remained stable between 2010 and 2017.
van der Ploeg et al. 2008	[23]	Longitudinal study	Australia	Proportion of children walked or cycled to school dropped from 57.7% to 25.5% between 1971 and 2003.
de Sá et al. 2015	[24]	Longitudinal study	São Paulo, Brazil	ASC rates among children decreased from 70% to 61% between 1997 and 2007.
Buliung, Mitra & Faulkner 2009	[25]	Longitudinal study	Greater Toronto Area, Canada	ASC rates among children decreased from 55.7% to 49.3% between 1986 and 2006.
Cui, Bauman & Dibley 2011	[26]	Longitudinal study	9 provinces in China	An increase in passive commuting to school was noted (from 3.6% in 1997 to 14.1%) between 1997 and 2006.
Yang et al. 2019	[27]	Modelling study	China	The decline in ASC as a global phenomenon.
2. How does ASC contribute to the physical activity levels of school children?				
Larouche et al. 2014	[30]	Systematic review	Not applicable	This systematic review aims to examine differences in physical activity, body composition and cardiovascular fitness between active and passive commuters. The majority of studies found that active school commuters were more active or that ASC interventions lead to increases in physical activity.
Lee, Orenstein & Richardson 2008	[31]	Systematic review	Not applicable	The review aims to examine the association between active commuting to school and outcomes of physical activity, weight,

Author and year of publication	Reference	Article type	Country/Region	Key / relevant findings
				and obesity in children. 32 studies were included. Most studies assessing physical activity outcomes found a positive association between ASC and overall physical activity levels.
Faulkner et al. 2009	[32]	Systematic review	Not applicable	This review aims to explore whether ASC is associated with higher physical activity level and healthier body weight. Nine out of thirteen studies demonstrated that children who actively commute to school accumulate significantly more physical activity.
Schoeppe et al. 2013	[33]	Systematic review	Not applicable	This review aims to review the associations of independent mobility and active travel to various destinations with physical activity, sedentary behaviour, and weight status. 52 studies published between January 1990 and March 2012 were included. Most studies showed significant positive associations between ASC and physical activity.
Cooper et al. 2005	[34]	Cross-sectional study	Denmark	In comparison with passive school commuting, walking to school was associated with 34 more minutes of daily MVPA in boys and 40 minutes in girls.
Sirard et al. 2005	[35]	Cross-sectional study	United States	ASC was associated with ~24 minutes more MVPA per day, with the difference being significant on weekdays only.<br>ASC was associated with 33% more accelerometer counts per minutes in the “before school” period and 27% more in the “after school” periods.
Goodman, Mackett & Paskins 2011	[36]	Cross-sectional study	United Kingdom	Each extra 1% of the day where the child engaged in ASC was associated with a 0.56% increase in daily minutes of MVPA.
Mendoza, Watson, Nguyen, et al. 2011	[37]	Cross-sectional study	United States	ASC was associated with greater average daily MVPA.
Denstel et al. 2015	[38]	Cross-sectional study	Australia, Brazil, Canada, China, Colombia, Finland, India, Kenya, Portugal, South Africa, the United Kingdom	Across all study sites, active school commuting was associated with 6.0 minutes more of weekday MVPA.

Author and year of publication	Reference	Article type	Country/Region	Key / relevant findings
			and the United States	
Saksvig et al. 2007	[39]	Cross-sectional study	United States	Girls who walked to and from school accumulated 13.7 more minutes of daily physical activity (including 4.7 minutes of MVPA). Those who walked only 1 way accumulated 2.5 more minutes of daily physical activity (including 2.2 minutes of MVPA) (Study recruited girls only).
van Sluijs et al. 2009	[40]	Cross-sectional study	United Kingdom	Walkers who lived 0.5–1.0 mile away from school accumulated 5.98 additional minutes of daily MVPA. Those who lived 1.0–5.0 miles away accumulated 9.77 more minutes of MVPA.
Cooper et al. 2010	[41]	Cross-sectional study	United Kingdom	Children who walked to school were more active than those who passively commuted during the whole day and the hour before school.
Mendoza et al. 2010	[42]	Cross-sectional study	United States	ASC was associated with higher daily MVPA.
King et al. 2011	[43]	Cross-sectional study	United Kingdom	ASC was significantly associated with total physical activity and MVPA
Panter et al. 2011	[44]	Cross-sectional study	United Kingdom	Children who commuted actively accumulated more of daily MVPA (3.3–6.9 minutes), with most of this difference occurring during the journey.
Owen et al. 2012	[45]	Cross-sectional study	United Kingdom	Children who commuted by car accumulated less MVPA (-7 min), total physical activity and steps per day (–813 steps) than active commuters.
Roth, Millett & Mindell 2012	[46]	Cross-sectional study	United Kingdom	Accelerometry results show that walkers and cyclists were more likely to be in the middle and highest physical activity tertiles.
Abbott et al. 2009	[47]	Cross-sectional study	Australia	Higher daily step counts in Year 5 boys (16,238 vs. 15,275) and girls (13,521 vs. 12,502) and in Year 10 girls (12,109 vs. 11,373). No differences in the other groups. In Year 5 and 10 girls, a dose-response association between the frequency of active school commuting and daily step counts.
McCormack et al. 2011	[48]	Cross-sectional study	Australia	Achieving the pedometer-based cut-points was positively associated with not being driven to school (OR 1.48).

Author and year of publication	Reference	Article type	Country/Region	Key / relevant findings
Loucaides & Jago 2008	[49]	Cross-sectional study	Cyprus	Children who walked to school accumulated significantly more steps in the "before school" (1355 vs. 983) and "after school" period (8026 vs. 6596) and in the whole day (14,710 vs. 12,795).
Duncan, Scott Duncan & Schofield 2008	[50]	Cross-sectional study	New Zealand	Girls who commuted actively to and from school accumulated 1052 additional daily steps. Girls who commuted actively only 1 way (e.g., to or from school) accumulated 834 more steps/day. (Study recruited girls only)
Hohepa et al. 2008	[51]	Cross-sectional study	New Zealand	Children who walked to and from school had higher step counts than those who walked only 1 way and those who were driven in cars (13,308 vs. 12,741 and 10,986 steps/day).
Larouche et al. 2011	[52]	Cross-sectional study	Canada	Active commuters accumulated 663 more steps per day (11,924 vs.11,261).
Johnson et al. 2010	[53]	Cross-sectional study	United States	Children who walked accumulated significantly more daily step count than those who were driven by car or bus (12,614 vs. 10,021 vs. 10,230).
Cuddihy, Davidson & Michaud-Tomson 2003	[54]	Cross-sectional study	Australia	Boys and girls who walked to school accumulated between 3227–3477 more steps/day than those who were driven by car or bus.
Murtagh & Murphy 2011	[55]	Cross-sectional study	Ireland	ASC was associated with higher daily step counts (16,118 vs. 13,363).
Tudor-Locke et al. 2002	[56]	Cross-sectional study	Russia	Omitting active school commuting was associated with a statistically significant lower prevalence of meeting different physical activity guidelines.
Heelan et al. 2005	[57]	Cross-sectional study	United States	ASC may contribute to the attainment of physical activity recommendations.<br>ASC was associated with more physical activity before school (9.0 vs. 6.4 min) than passive school commuting.
Trang et al. 2009	[58]	Cross-sectional study	Vietnam	Passive school commuting was associated with greater odds of not meeting physical activity guidelines in boys (OR = 7.4) and girls (OR = 2.6).

Author and year of publication	Reference	Article type	Country/Region	Key / relevant findings
Daly-Smith et al. 2010	[59]	Cross-sectional study	United Kingdom	Each additional day where children engaged in active school commuting was associated with a greater likelihood of meeting physical activity guidelines (OR = 2.52-6.45).
Rosenberg et al. 2006	[60]	Longitudinal study	United States	No association between ASC and physical activity at follow-up. At baseline, boys who actively commuted were more active.
Carver et al. 2011	[61]	Longitudinal study	Australia	There were no significant associations between active school commuting and MVPA among younger boys and girls (aged 5-6 yrs) at baseline and follow-ups.
Andersen et al. 2011	[62]	Longitudinal study	Denmark	At baseline, boy cyclists were more active than non-cyclist. At 6-year follow-up, girl cyclists were more active than non-cyclist.
Camiletti-Moirón et al. 2020	[63]	Longitudinal study	Spain	Active school commuting was positively associated with MVPA at 1-year follow-up.
Alwis et al. 2007	[64]	Longitudinal study	Sweden	No differences between active and passive commuters in minutes of daily MVPA and VPA, neither at baseline, nor at follow-up.
Löfgren et al. 2010	[65]	Longitudinal study	Sweden	No differences between active and passive commuters in minutes of daily MVPA and VPA, neither at baseline, nor at follow-up.
Smith et al. 2012	[66]	Longitudinal study	United Kingdom	Compared to children whose reported mode of travel did not change, a change from a passive to an active mode of commuting was associated with an increase in daily minutes spent in MVPA. This increase represented 12% of boys' and 13% of girls' total daily time spent in MVPA at follow-up.
Heelan et al. 2009	[67]	Interventional study (Walking school bus)	United States	Participants from the intervention school accumulated more daily physical activity (78.01 vs. 60.62 min). Irrespective of the school, frequent walkers were more active than infrequent walkers and passive travelers (80.61 vs. 70.48 vs. 60.70 min/day).
Sirard et al. 2008	[68]	Interventional study (Walking school bus)	United States	Participants assigned to the walking school bus increased their level of physical activity (from 1,052.9–2,169.3 cpm) and accumulated 14 additional minutes of MVPA during the 45 minutes before school during commute.
Mendoza, Watson, Baranowski, et al. 2011	[69]	Interventional study (Walking school bus)	United States	Children in the experimental group increased daily MVPA from 46.6 to 48.8 minutes; those from the control group decreased from 46.1 to 41.3 minutes. Boys were more likely to increase their MVPA

Author and year of publication	Reference	Article type	Country/Region	Key / relevant findings
Cooper et al. 2006	[70]	Cross-sectional study	Denmark	Participants who walked to school recorded consistently higher values for MVPA than those using passive travel or cycling, but differences were not statistically significant.
Voss & Sandercock 2010	[71]	Cross-sectional study	United Kingdom	Children who cycled to/from school had significantly higher mean physical activity scores than those who used other modes of transport.
Cooper et al. 2003	[72]	Cross-sectional study	United Kingdom	Boys who walked were more active (813.3 vs. 647.3 accelerometer counts per minutes) accumulating 45 additional minutes of MVPA on weekdays and 22 on weekend days.
Metcalf et al. 2004	[73]	Cross-sectional study	United Kingdom	Physical activity level during to and from school journeys was significantly higher among those who walked than those who travelled by car
Dollman & Lewis 2007	[74]	Cross-sectional study	Australia	ASC has no significant association with free-time physical activity.
Ford et al. 2007	[75]	Cross-sectional study	United Kingdom	Children who walked to/from school were more active during the hour before and after school (983.2 vs. 762.7 accelerometer counts per minute); no difference in other time periods.
Baig et al. 2009	[76]	Cross-sectional study	United Kingdom	Walking to and from school was not significantly associated with children's physical activity during school time.
Wen et al. 2010	[77]	Cross-sectional study	Australia	No association between ASC and physical activity level during non-school hours.
3. *What are the health benefits of ASC?*				
Larouche et al. 2014	[30]	Systematic review	Not applicable	This systematic review aims to examine differences in physical activity, body composition and cardiovascular fitness between active and passive commuters. There is conflicting evidence regarding the associations between ASC and body composition indicators.
Lee, Orenstein & Richardson 2008	[31]	Systematic review	Not applicable	The review aims to examine the association between active commuting to school and outcomes of physical activity, weight, and obesity in children. 32 studies were included. There might be no association between active commuting and reduced weight or BMI.

Author and year of publication	Reference	Article type	Country/Region	Key / relevant findings
Faulkner et al. 2009	[32]	Systematic review	Not applicable	This review aims to explore whether ASC is associated with higher physical activity level and healthier body weight. Evidence for the impact of ASC in promoting healthy body weights for children is not compelling.
Schoeppe et al. 2013	[33]	Systematic review	Not applicable	This review aims to review the associations of independent mobility and active travel to various destinations with physical activity, sedentary behaviour, and weight status. 52 studies published between January 1990 and March 2012 were included. An inverse relationship between active travel to school and weight status was evident, but findings were inconsistent.
Mendoza, Watson, Nguyen, et al. 2011	[37]	Cross-sectional study	United States	ASC was associated with lower sum of triceps and subscapular skinfolds. The association with waist circumference was not significant.
Rosenberg et al. 2006	[60]	Longitudinal study	United States	At baseline, boys who actively commuted had lower sum of triceps and calf skinfolds. No differences were noted in girls. No differences in the changes in skinfolds at 2-year follow-up.
Camiletti-Moirón et al. 2020	[63]	Longitudinal study	Spain	Active school commuting volume at baseline had no total effect on BMI, waist circumference, physical fitness, handgrip test and standing long jump at 2-year follow-up.
Masoumi 2017	[78]	Systematic review	Not applicable	This review aims to explore the relationship between ASC and BMI in children. 13 papers were included. No conclusive result can be derived from studies on ASC and BMI of children.
Xu, Wen & Rissel 2013	[79]	Systematic review	Not applicable	This review aims to examine the relationships between active transport to work or school and cardiovascular health, body weight. 6 studies involving school children were included. The evidence for the impact of ASC on body weight was not consistent.
Lubans et al. 2011	[80]	Systematic review	Not applicable	This review aims to explore the relationship between ASC and health-related fitness among youth. 27 articles were included. There is some evidence to suggest that ASC is associated with a healthier body composition.
Ruiz-Hermosa et al. 2018	[81]	Cross-sectional study	Spain	There were no differences in BMI, waist circumference, adiposity as measured by bioimpedance analysis system, and cardiovascular

Author and year of publication	Reference	Article type	Country/Region	Key / relevant findings
				fitness as measured by 20-metre shuttle run test, between active commuters and nonactive commuters.<br>No differences were found between walking to school and passive commuters with Nonverbal Intelligence and General Intelligence outcomes in children aged 4 to <6 years old. Walking to school was not associated with Logical Reasoning, Spatial Factor and General Intelligence outcomes in children aged ≥6 to 7 years old.
Mwaikambo et al. 2015	[82]	Cross-sectional study	Tanzania	Children who walked to and from school were less likely to be overweight or obese than those who used vehicles.
Manyanga et al. 2020	[83]	Cross-sectional study	Mozambique	Passive school commuting was associated with overweight/obesity.
Silva & Lopes 2008	[84]	Cross-sectional study	Brazil	ASC was associated with a lower prevalence of excess body fat as estimated from the triceps skinfold.
Roth, Millett & Mindell 2012	[46]	Cross-sectional study	United Kingdom	There was no difference in the risk of being obese in children who walked and cycled to school.
Voss & Sandercock 2010	[71]	Cross-sectional study	United Kingdom	There was no association between travel mode and BMI.
Panter et al. 2010	[85]	Cross-sectional study	United Kingdom	No statistically significant association was observed between BMI category and travel mode to school.
Andegiorgish et al. 2012	[86]	Cross-sectional study	China	There was no difference in the risk of being overweight in children who walked and cycled.
Ostergaard et al. 2013	[87]	Cross-sectional study	Norway	No association were found between different modes of transport to school and BMI.<br>Male cyclists have higher cardiovascular fitness than both walkers and passive commuters. Cardiovascular fitness did not differ between walkers and passive commuters neither when comparing across gender nor when comparing across age groups.<br>Muscle endurance measured as back extension duration in seconds was significantly longer in children walking and cycling to/from school compared to passive. There was no statistical difference between cycling and walking children.

Author and year of publication	Reference	Article type	Country/Region	Key / relevant findings
Larouche et al. 2011	[52]	Cross-sectional study	Canada	Active commuters had lower waist circumference (69.1 vs. 72.2 cm), but this difference was not significant.
Andersen et al. 2011	[62]	Longitudinal study	Denmark	There was also no statistically significant difference between cyclists and non-cyclists in waist circumference.<br>Cyclists have better CVD risk factor profiles.
Silva et al. 2011	[88]	Cross-sectional study	Brazil	No differences in waist circumference between active and passive school commuters. Active school commuters were more likely to be classified as “fit” based on their performances on the 20m shuttle run test.
Noonan et al. 2017	[89]	Cross-sectional study	United Kingdom	Active school commuters had significantly higher waist circumference than passive school commuters. No associations between school travel mode and fitness.
Sun, Liu & Tao 2015	[90]	Cross-sectional study	China	Children who commuted via active modes had lower waist circumference scores points than those who used passive transport. Active school commuting was associated with .907 lower) odds of having depressive symptoms compared with passive school commuting.
Klein-Platat et al. 2005	[91]	Cross-sectional study	France	Girls who reported no active school commuting had lower waist circumference (65 vs. 65.9–66.5 cm). There was no difference in waist circumference in boys.
Alwis et al. 2007	[64]	Longitudinal study	Sweden	No difference in DEXA-measured percentage body fat at baseline, and no difference in annual changes.<br>There were no differences in baseline or annual changes in BMC or bone width when the transportation groups were compared.
Löfgren et al. 2010	[65]	Longitudinal study	Sweden	No difference in DEXA-measured percentage body fat at baseline, and no difference in annual changes.<br>There were no differences in the annual changes in BMC or bone width when comparing girls or boys who walked or cycled to school with those who went by car or bus.
Smith, Aggio & Hamer 2017	[92]	Cross-sectional study	United Kingdom	No associations between school travel mode and adiposity as measured by bioimpedance analysis system, and other health

Author and year of publication	Reference	Article type	Country/Region	Key / relevant findings
				markers (grip strength, horizontal jump test, flexibility and peak flow meter).
Ford et al. 2007	[75]	Cross-sectional study	United Kingdom	No association between school travel mode and fat mass as measured by air displacement plethysmography with school commuting mode.
Cooper et al. 2006	[70]	Longitudinal study	Denmark	At 6-year follow-up, cyclist has significantly higher cardiovascular fitness than non-cyclists.
Cooper et al. 2008	[93]	Longitudinal study	Denmark	At 6-year follow-up, cardiovascular fitness was consistently higher in cyclists (mean difference = 1.1–15.2% depending on gender and age group) than in walkers or passive commuters.
Chillón et al. 2012	[94]	Longitudinal study	Sweden	Children who bicycled to school increased their fitness 13% more than those who used passive modes and 20% more than those who walked. Children who used passive modes or walked at baseline and bicycled to school at 6 years later increased their fitness 14% more than those who remained using passive modes or walking at follow-up. Bicycling to school in childhood was related to improvements in fitness 6 years later.
Chillón et al. 2010	[95]	Cross-sectional study	Sweden, Estonia	Higher cardiovascular fitness was significantly associated with cycling to school in children of both sexes. Longitudinal regression models showed that a change in travel mode from non-cycling to cycling was a significant predictor of cardiovascular fitness at follow-up after adjustment for potential confounders. Participants who did not cycle to school at baseline, but who had changed to cycling at follow-up, were significantly fitter than those who did not cycle to school at either time point, a difference of 9%.
Børrestad et al. 2012	[96]	Interventional study	Norway	At 12-week follow up, a significant difference between those starting cycling and those who did not starting cycling was observed in cardiovascular fitness.
Voss & Sandercock 2010	[71]	Cross-sectional study	United Kingdom	Walkers and cyclists of both sexes were significantly fitter than passive transport users as measured by 20 - metre shuttle run test.
Muntaner-Mas et al. 2018	[97]	Cross-sectional study	Spain	Fitness was not associated with the mode of commuting in children.

Author and year of publication	Reference	Article type	Country/Region	Key / relevant findings
Meron et al. 2011	[98]	Cross-sectional study	Australia	In the 2010 cohort, participants who made frequent car trips were more likely to be in the least fit quartile based on the 20m shuttle run test. No differences were found in the 2004 cohort.
Aires et al. 2011	[99]	Cross-sectional study	Portugal	Active school commuting was associated with greater cardiovascular fitness as measured by 20-metre shuttle run test. Time spent engaging in ASC was not significantly associated with cardiovascular fitness.
Sandercock & Ogunleye 2012	[100]	Cross-sectional study	United Kingdom	The multivariate odds of being fit were higher in active commuting group than in passive commuting groups.
Villa-González, Ruiz & Chillón 2015	[101]	Cross-sectional study	Spain	No associations between active commuters and health-related fitness (20-metre shuttle-run test, push-up test, handgrip test, standing long jump, leg extension test)
Cohen et al. 2014	[102]	Cross-sectional study	United Kingdom	Cyclists had greater handgrip strength than passive commuters. Vertical jump height was greater in walkers and cyclists compared with passive commuters. Jump peak power was also higher in walkers than in the passive travel group.
Pires et al. 2017	[103]	Cross-sectional study	Brazil	Girls who actively commute to school showed better levels of upper limb strength and velocity. No significant difference was observed for the physical fitness between commute groups in boys.
Henriques-Neto et al. 2020	[104]	Systematic review	Not applicable	This review aims to examine the relationship between active commuting and physical fitness. 11 studies on children and adolescents were included. Active commuting is inconsistently related to physical fitness.
López-Vicente et al. 2016	[105]	Cross-sectional study	Spain	No differences were found between active commuting to school and passive commuters in Working Memory and Attention outcomes. Active commuting for more than 50 min was associated with better 3-back scores at baseline, as compared with passive commuting.
Westman et al. 2016	[106]	Cross-sectional study	Sweden	Active commuting was not associated with scores on the word fluency task
Fang & Lin 2016	[107]	Cross-sectional study	Taiwan	Active commuting to school was positively related to the number of objects, correctness of route orientation and aggregated scores,

Author and year of publication	Reference	Article type	Country/Region	Key / relevant findings
				and negatively associated with correctness of route structure for the spatial cognition maps of the participants. Active commuting to school was not associated with the number of landmarks, paths and places in the participants' cognitive maps.
Moran, Eizenberg & Plaut 2017	[108]	Cross-sectional study	Israel	The accuracy scores obtained from maps of children who walk to school most of the week were significantly higher than those of children who did not. The richness scores of the sketch maps did not differ according to the children's school travel mode.
Rissotto & Tonucci 2002	[109]	Cross-sectional study	Italy	Children who walk to school on their own achieved the best performance both in drawing the sketch map of the itinerary and in sketching the route on a blank map of the quarter.
Haapala et al. 2014	[110]	Cross-sectional study	Finland	Active school commuting was positively associated with reading fluency and reading comprehension, but not with mathematics-related skills in boys. In girls, no associations were found.
Domazet et al. 2016	[111]	Cross-sectional study	Denmark	Bicycling to school was associated with superior mathematics performance compared to passive transportation, and walking to school was not associated with mathematics performance.
García-Hermoso et al. 2017	[112]	Cross-sectional study	Chile	Active commuting to school was not associated with higher scores in any grades after adjustment for potential confounders. Adjusted binary logistic regression analysis suggested that adolescents who spent between 30 and 60 min actively commuting were more likely to obtain high academic achievement in language and mathematics.
Mora-Gonzalez et al. 2017	[113]	Cross-sectional study	Spain	Passive primary school commuters had better grades in maths, Spanish, English, natural sciences and grade point average than active commuters.
Stark et al. 2018	[114]	Cross-sectional study	Austria	Active school commuting is positively associated with children's psychological well-being; Parents reported strong positive associations between active commuting modes and the well-being of their children.
Pizarro et al. 2013	[115]	Cross-sectional study	Portugal	Walkers have higher odds to have better high-density lipoprotein cholesterol profiles than non-active commuters, independent of

Author and year of publication	Reference	Article type	Country/Region	Key / relevant findings
				moderate-to-vigorous physical activity. No associations were found for other metabolic risk factors.
Gutiérrez-Zornoza et al. 2015	[116]	Cross-sectional study	Spain	No differences were found between children who daily walked/cycled to school and those commuting actively to school less frequently in metabolic syndrome index.
Zhang et al. 2020	[117]	Cross-sectional study	United Kingdom	There were no significant differences between active and passive school commuters in systolic and diastolic blood pressure, and lung function.
4. *What are the determinants of ASC behaviours?*				
Tudor-Locke, Ainsworth & Popkin 2001	[7]	Opinion paper	Not applicable	Proposed ASC as a way to promote physical activity among children.
Ginja et al. 2018	[118]	Review	Not applicable	The paper reviewed the various frameworks on ASC grounded in the Behavioural Ecological Model.
Lu et al. 2014	[119]	Systematic review	Not applicable	This review aims to investigate perceived barriers to children's ACS.<br>39 papers were included. A number of perceived barriers (personal, physical environmental, and social environmental) were found to be associated with ASC.
McMillan 2005	[120]	Review	Not applicable	This paper introduced the McMillan's framework of Elementary-Aged Child's Travel Behaviour, which emphasised the role of parents as the principal decision-makers regarding the active transport behaviour of children.
Pont et al. 2009	[121]	Systematic review	Not applicable	This review investigates the environmental (physical, economic, socio-cultural and political) correlates of active commuting among young people aged 5–18 years. 38 studies were included. Greater distance, increasing household income and increasing car ownership are consistently associated with lower rates of active commuting among children.
D'Haese et al. 2015	[122]	Systematic review	Not applicable	This review aims to determine the relationship between a wide range of

Author and year of publication	Reference	Article type	Country/Region	Key / relevant findings
				physical environmental characteristics and different contexts of active commuting in 6- to 12-year-old children across different continents. 65 papers were included. ASC was positively associated with walkability. Walking to school was positively associated with walkability, density and accessibility. Evidence for a possible association was found for traffic safety and all forms of active transportation to school.
Wangzom, White & Paay 2023	[123]	Systematic review	Not applicable	This review aims to investigate mediating factors influencing children's ASC. 65 papers were included. Perceived traffic safety, neighbourhood safety, and distance to school were found to be key mediating factors influencing children's active commuting to school.
Aranda-Balboa et al. 2020	[124]	Systematic review	Not applicable	This review aims to investigate parental barriers to ASC. 27 papers were included. The main parental barriers to ASC reported by parents of children were built environment, traffic safety, distance, crime-related safety and social support.
Rothman et al. 2018	[125]	Systematic review	Not applicable	This review aims to investigate factors related to ASC in North America. 63 papers from North America were included. Distance to school was most strongly associated with ASC. Individual, parental and societal correlates had moderate positive associations with ASC including: child age, lower parental education, income and other income related factors, race and positive perceptions of ASC.
Wong, Faulkner & Buliung 2011	[126]	Systematic review	Not applicable	This review aims to examine and summarise the relationships between objectively measured built environment features and ASC. Only distance was consistently found to be negatively associated with ASC. Consistent findings of positive or negative associations were not found for land use mix, residential density, and intersection density.

Author and year of publication	Reference	Article type	Country/Region	Key / relevant findings
Ikeda et al. 2018	[127]	Systematic review	Not applicable	This review aims to identify the associations between school travel modes in children aged 5–13 years and perceived physical environments as well as social and sociodemographic characteristics. 37 studies were included. ASC was associated positively with safety, walkability and neighbourhood social interactions, and negatively with travel distance and car ownership.
Nikitas, Wang & Knamiller 2019	[128]	Qualitative study	United Kingdom	Focus group sessions with parents revealed 6 critical thematic areas that explain the decision-making behind potential walking school bus uptake, namely, logistics, safety, trust, health and wellbeing, emotional needs and educational opportunities.
Forsberg et al. 2020	[129]	Qualitative study	Sweden	Interview to explore parents' attitudes and beliefs towards ASC have identified one main theme "Parenting and active school transportation – making route choices in a changed landscape" and four subthemes, "Knowing that it is beneficial while struggling with daily life", "Considering barriers and solutions to enable AST", "Parenting is challenging and about balancing", and "Reflecting and contemplating about what we and others do".
O'Connor & Brown 2013	[130]	Qualitative study	Australia	In depth individual and focus group interviews with parents revealed that parents were aware of the consequences of their decisions regarding their children's mode of commuting to school. The decision-making involves constant negotiation between a desire to allow their child independence and the question 'what if?'.
Ahern et al. 2017	[131]	Qualitative study	United Kingdom	Semi-structured interviews with parents revealed that distance was the biggest barrier to ASC. Time constraints were reported as the main barrier to parents accompanying children in ASC, while concerns about safety deterred parents from allowing children to travel independently.
Faulkner et al. 2010	[132]	Qualitative study	Canada	Semi-structured interviews with parents revealed that decision on mode of school commuting appeared to be primarily influenced by concerns about traffic, the child's personal safety, and the child's

Author and year of publication	Reference	Article type	Country/Region	Key / relevant findings
				maturity and cognitive ability regarding navigating his/her way to/from school safely. Following the escort decision, parents considered mode choice, typically selecting what they perceived to be the easiest and most convenient way to travel. The ascription of convenience to the various modes of transportation was influenced by perceptions of travel time and/or distance to/from school.
Wilson, Clark & Gilliland 2018	[133]	Cross-sectional study	Canada	There are significant differences in how parents and children perceive barriers to active school commuting. Parent perceptions of barriers are found to have a greater influence on children's active school travel behaviour than children's perceptions.
Morris et al. 2022	[134]	Qualitative synthesis	Not applicable	Synthesis of qualitative and ethnographic studies of children's experiences of socio-material environments on their school journey showed that children can feel vulnerable, but also negotiate journeys and manage risks, enjoy shared and solitary mobility, and explore their material environments. School journeys offer children a place to learn and develop agency within their socio-material environments.
Zhu & Lee 2009	[135]	Cross-sectional study	United States	Negative correlates of ASC included school bus availability.
Zhu, Arch & Lee 2008	[136]	Cross-sectional study	United States	School bus service reduced the likelihood of walking to school. Parents consider safety to be another important factor in choosing walking as their children's school transportation mode. The "perception" of safety shows no significant differences among schools, although the factual crime or crash data showed dramatic differences. This finding implies that parents may overexaggerate dangers from traffic and crime when it comes to children's school travel.
Gustat et al. 2015	[137]	Cross-sectional study	United States	Predictors of walking or cycling to school include parental perception of school encouragement of walking or cycling.
Ahlport et al. 2008	[138]	Qualitative study	United States	Focus group studies with parents revealed that encouragement of ASC by school is a facilitator of ASC.

Author and year of publication	Reference	Article type	Country/Region	Key / relevant findings
Rothman et al. 2015	[139]	Cross-sectional study	Canada	Parents' perception of danger is not always in accordance with objective measures of traffic danger.
Marzi et al. 2020	[140]	Systematic review	Not applicable	This review aims to evaluate the effects of ASC interventions on girls and<br>Boys. ASC is less prevalent in girls than in boys, with boys more likely to cycle to school while girls prefer walking
Sirard & Slater 2008	[141]	Review	Not applicable	There are conflicting results on the association between ASC and the age of the child, with most studies demonstrating null effect.
Zuniga 2012	[142]	Qualitative study	United States	Interview with parents on their mode choice decision revealed two contrasting themes: barrier elimination and barrier negotiation. Regular ASC appears to diminish parents' perceptions of barriers so that negotiation becomes second nature.
5. *What interventions are effective in promoting ASC?*				
Tudor-Locke, Ainsworth & Popkin 2001	[7]	Opinion paper	Not applicable	This paper introduces the intervention "Safe Routes to School (SRTS)".
Smith et al. 2015	[144]	Systematic review	Not applicable	This review aims to summarise effectiveness of Walking school buses (WSBs) interventions. 12 studies were included. WSBs were found to be associated with increased prevalence of walking to school and general activity levels although not always significantly. Time constraints emerged as barriers to WSBs, impacting on recruitment of volunteers and children to the WSBs. Facilitators of WSBs included children enjoying socializing and interacting with the environment.
Chillón et al. 2011	[146]	Systematic review	Not applicable	This review aims to analyse intervention studies related to ASC. 14 studies published through 2010 were included. Most of the interventions reported a small effect size on ASC.
Pang, Kubacki & Rundle-Thiele 2017	[147]	Systematic review	Not applicable	This review aims to analyse ASC interventions. 18 studies published between 2010 and 2016 were included. The analysis of the interventions using the ALBD Community Action Model showed that Preparation and Promotion were used much more frequently than Policy and Physical projects. The methodological

Author and year of publication	Reference	Article type	Country/Region	Key / relevant findings
				quality 14 out of 18 included interventions were assessed as weak according to the EPHPP framework.
Larouche et al. 2018	[148]	Systematic review	Not applicable	This review aims to assess effectiveness of interventions in increasing<br>ASC. 27 articles reporting 30 interventions, published between February 1, 2010 and October 15, 2016, were included. These interventions may increase ASC among children; however, there was substantial heterogeneity across studies and quality of evidence remains low.
Villa-González et al. 2018	[149]	Systematic review	Not applicable	This review aims to assess the effectiveness of interventions focused on ASC. 23 intervention studies published from February 2010 to December 2016 were identified. Most interventions reported a small effect size on active commuting to school. The quality assessment was rated as weak in most studies.
Jones et al. 2019	[150]	Systematic review	Not applicable	This review aims to assess the effectiveness of active school commuting interventions. Seventeen studies were included. Effectiveness assessment found a statistically significant difference in ASC outcomes in favour of the intervention. Walking school buses and educational strategies are most effective for increasing relevant outcomes, although overall study quality was weak.
Schönbach et al. 2020	[151]	Systematic review	Not applicable	This review aims to summarize the evidence on strategies and effects of school-based interventions focusing on increasing active school transport by bicycle. Nine studies investigating seven unique interventions performed between 2012 and 2018 were included. There is an indication that a bicycle train to/from school among children in primary school is a promising intervention. All studies were rated as weak quality.
Jacob et al. 2021	[152]	Systematic review	Not applicable	This review aims to synthesise the economic evidence for the cost and benefit of infrastructure and non-infrastructure programs that improve the safety, convenience, and attractiveness of ASC. 9 economic evaluation studies were included. Overall, these

Author and year of publication	Reference	Article type	Country/Region	Key / relevant findings
				programs were found to generate societal economic benefits that exceed the societal cost.
6. *What are the advancements in research techniques related to ASC?*				
Wong, Faulkner & Buliung 2011	[126]	Systematic review	Not applicable	This review discusses the use of geographic information system methodologies used in objectively measurement of built environment features in ASC research.
Jacobs et al. 2021	[153]	Cross-sectional study	Australia	This study made use of geospatial datasets from government sources to objectively measure environmental features like recreational facilities, greenspaces and walkability.
Merom et al. 2006	[154]	Cross-sectional study	Australia	This study measured home-to-school distance by asking parents to estimate it to the nearest 100m.
Bringolf-Isler et al. 2008	[155]	Cross-sectional study	Switzerland	In this study, home-to-school distance was calculated as a straight-line distance using geographic information system based on the home and school address.
Panter et al. 2010	[85]	Cross-sectional study	United Kingdom	In this study, the shortest route and distance between home and school was calculated for each participant using ArcGIS Network Analyst.
Macdonald et al. 2019	[156]	Cross-sectional study	United Kingdom	In this study, the shortest route and distance between home and school was calculated for each participant using Google Maps.
Ikeda et al. 2019	[157]	Cross-sectional study	New Zealand	This study recorded the actual school commuting route by asking the child to draw their routes on an electronic map.<br>This study utilised structural equation modelling.
Dessing et al. 2014	[158]	Cross-sectional study	The Netherlands	This study recorded the actual school commuting route using wearable GPS devices.
Villa-González et al. 2019	[159]	Cross-sectional study	Spain	This study recorded the actual school commuting route using wearable GPS devices.
Mah et al. 2017	[163]	Cross-sectional study	Canada	In this study, parental perceived neighbourhood traffic and crime safety were assessed using the Neighborhood Environmental Walkability Scale-Youth instrument.
Ozbil et al. 2021	[164]	Cross-sectional study	Turkey	In this study, parental perception of neighbourhood environment (accessibility and streets, access to urban services, architecture,

Author and year of publication	Reference	Article type	Country/Region	Key / relevant findings
				green spaces, safety, and maintenance) was measured using the Neighborhood Environment Walkability Scale instrument.
Larouche et al. 2015	[165]	Cross-sectional study	Australia, Brazil, Canada, China, Colombia, Finland, India, Kenya, Portugal, South Africa, United Kingdom, United States	In this study, children's perception of neighbourhood environment was measured using the Neighborhood Environmental Walkability Scale-Youth instrument.
Van Kann et al. 2014	[166]	Cross-sectional study	the Netherlands	In this study, children's perception of neighbourhood environment was measured using the Neighborhood Environment Walkability Scale instrument.
Balboa et al. 2022	[167]	Validation study	Spain	A validation study to validate the Mode and Frequency of Commuting To and From School Questionnaire in Spanish children and parents.
Chillón et al. 2017	[168]	Validation study	Spain	A validation study to validate the Mode and Frequency of Commuting To and From School Questionnaire in Spanish youths.
Aranda-Balboa et al. 2020	[169]	Validation study	Spain	A validation study to validate Family Commuting-to-School Behaviour Questionnaire among Spanish parents and children.
Lu et al. 2014	[119]	Systematic review	Not applicable	This review commented that *"the lack of theoretical basis might account for the overarching number of exploratory studies among the reviewed studies, which typically assume only their direct effects on active commuting to school without considering interaction among predictor variables."*
Easton & Ferrari 2015	[170]	Cross-sectional study	United Kingdom	This study commented on the *"importance of taking the sociospatial clustering of individual pupils into account in any examination of school travel patterns. Studies such as those using survey sample data that do not group pupils into neighbourhoods and schools may miss these higher-*

Author and year of publication	Reference	Article type	Country/Region	Key / relevant findings
				level effects and may have led to the over-estimation of the proportion of variance explained by individual and urban form variables." This study utilised cross-classified multilevel model.
Ginja et al. 2018	[118]	Review	Not applicable	The paper reviewed the various frameworks on ASC grounded in the Behavioural Ecological Model.
Bosch et al. 2020	[171]	Cross-sectional study	United Kingdom	This study utilised multilevel ordered logistic regression modelling.
Aarts et al. 2013	[172]	Cross-sectional study	The Netherlands	This study utilised Multilevel multinomial logistic regression analyses.
Trapp et al. 2012	[173]	Cross-sectional study	Australia	This study used a multilevel ecological framework to investigate individual, social, and environmental factors associated with walking to and from school among elementary school-aged children.
Moodie et al. 2009	[174]	Economic analysis	Australia	This study used logic pathway to model the effects on body mass index and disability-adjusted life years of the Victorian Walking school bus program if applied throughout Australia.
Yang & Diez-Roux 2013	[176]	Modelling study	United States	In this study, an agent-based model was developed to simulate children's school travel behaviour within a hypothetical city.
Yang et al. 2014	[177]	Modelling study	United States	In this study, an agent-based model was used to examine the impact of the walking school bus (WSB) on children's active travel to school.
Jing, You & Chen 2018	[178]	Modelling study	United States	In this study, an agent-based model was used to simulate the mode choice decision of parents based on distance, traffic safety and social influence.
Almagor et al. 2021	[179]	Modelling study	United States	This study explores the potential impact of interventions on physical activity by using an agent-based model simulating children's daily activities (including school commuting) in an urban environment.
7. What are the current limitations in ASC research?				
Herrador-Colmenero et al. 2014	[180]	Systematic review	Not applicable	This systematic review of self-reported measures used for assessing mode and frequency of commuting to and from school in

Author and year of publication	Reference	Article type	Country/Region	Key / relevant findings
				youngsters. It commented that *"the self-report measures used in the literature for assessing commuting to school tend to be heterogeneous and make difficult interstudies comparisons."*
Larouche et al. 2014	[30]	Systematic review	Not applicable	This systematic review aims to examine differences in physical activity, body composition and cardiovascular fitness between active and passive commuters. It commented that *"the absence of significant differences in several studies could be due to … the lack of analyses differentiating between walking and cycling"* and *"some studies classified children using AST only 1 day per week as active travelers. This can potentially bias the results toward the null hypothesis."*
Goodman, Mackett & Paskins 2011	[36]	Cross-sectional study	United Kingdom	In this study, minutes spent in ASC in one week was used as an independent variable. The result shows a dose-response relationship with daily total MVPA.
Daly-Smith et al. 2010	[59]	Cross-sectional study	United Kingdom	In this study, days of active commuting in one week was used as an independent variable. The result shows a dose-response relationship with the odds of meeting physical activity recommendations.
Abbott et al. 2009	[47]	Cross-sectional study	Australia	In this study, participants were classified based on their frequency of walking to school in a week (non-walker, walked 5 times or less a week, walked 6 times or more a week). The result shows a dose-response relationship with daily step count.
Schoeppe et al. 2013	[33]	Systematic review	Not applicable	This review aims to review the associations of independent mobility and active travel to various destinations with physical activity, sedentary behaviour, and weight status. 52 studies were included.
Faulkner et al. 2009	[32]	Systematic review	Not applicable	This review aims to explore whether ASC is associated with higher physical activity level and healthier body weight. It commented that *"Very young children may live closer to their elementary schools, or be only allowed to walk to school when the school is located close to home, or if a parent, guardian or sibling accompanies them to school."*

Author and year of publication	Reference	Article type	Country/Region	Key / relevant findings
Masoumi 2017	[78]	Systematic review	Not applicable	This review aims to explore the relationship between ASC and BMI in children. It commented that *"Further research by means of larger samples in less-studied contexts and cultures may be useful for shaping the overall structure of the subject."*
Pang, Kubacki & Rundle-Thiele 2017	[147]	Systematic review	Not applicable	This review commented that *"Further, all of the 18 interventions were carried out in developed countries, yet physical inactivity among children is a significant challenge in many developing countries suggesting there is an opportunity to extend active school transportation intervention testing geographically."*, *"Fourteen out of 18 studies were assessed as weak. Notably, selection biases, lack of double blinding, and not controlling for confounders were key issues identified"* and *"our results show that theory testing and building remains limited in AST (research)."*
D'Haese et al. 2015	[122]	Systematic review	Not applicable	This review identified that continent-specific associations were found for ASC determinants. For example, general safety and traffic safety were associated with ASC in North America and Australia but not associated with ASC in Europe.
Larouche et al. 2018	[148]	Systematic review	Not applicable	This systematic review of ASC interventions commented that *"Study quality was rated as weak for 27/30 interventions (due notably to lack of blinding of outcome assessors, unknown psychometric properties of measurement tools, and limited control for confounders)"*, *"most interventions did not include an explicit theoretical framework"*, and suggested that future studies should *"examine potential moderators and mediators of travel behavior change to help refine current interventions."*
Lubans et al. 2011	[80]	Systematic review	Not applicable	This review aims to explore the relationship between ASC and health-related fitness among youth. 27 articles were included.
Xu, Wen & Rissel 2013	[79]	Systematic review	Not applicable	This review aims to examine the relationships between active transport to work or school and cardiovascular health, body weight. 6 studies involving school children were included.
Lee, Orenstein & Richardson 2008	[31]	Systematic review	Not applicable	The review aims to examine the association between active commuting to school and outcomes of physical activity, weight, and obesity in children. 32 studies were included.

**Author and year of publication**	**Reference**	**Article type**	**Country/Region**	**Key / relevant findings**
Henriques-Neto et al. 2020	[104]	Systematic review	Not applicable	This review aims to examine the relationship between active commuting and physical fitness. 11 studies on children and adolescents were included.
Ruiz-Hermosa et al. 2019	[182]	Systematic review	Not applicable	The review aims to evaluate the link between ASC and cognitive performance and academic achievement in children and adolescents. Only 5 studies involving school children were identified.
Phansikar et al. 2019	[183]	Systematic review	Not applicable	This review aims to synthesize literature on the relationship between ASC and cognition or academic achievement. Only 8 studies involving school children were identified.
Morris et al. 2022	[134]	Systematic synthesis	Not applicable	This paper uses meta-ethnography to synthesise qualitative and ethnographic studies of children's (aged 5–13) experiences of socio-material environments on their school journey. 18 studies were included.
Villa-González et al. 2018	[149]	Systematic review	Not applicable	This review aims to assess the effectiveness of interventions focused on ASC. 23 intervention studies published from February 2010 to December 2016 were identified. It commented that *"most studies (21/23) were rated as weak in the quality of the study components. The main reasons were low scores (i.e., weak rating) in selection bias, study design, control of confounders, and data collection components."*
Chillón et al. 2011	[146]	Systematic review	Not applicable	This review commented that *"the studies generally failed to describe their theoretical frameworks"*.
